# Type 2 Diabetes Monocyte MicroRNA and mRNA Expression: Dyslipidemia Associates with Increased Differentiation-Related Genes but Not Inflammatory Activation

**DOI:** 10.1371/journal.pone.0129421

**Published:** 2015-06-17

**Authors:** Lucy Baldeón R., Karin Weigelt, Harm de Wit, Behiye Ozcan, Adri van Oudenaren, Fernando Sempértegui, Eric Sijbrands, Laura Grosse, Anton-Jan van Zonneveld, Hemmo A. Drexhage, Pieter J. M. Leenen

**Affiliations:** 1 Department of Immunology, Erasmus MC, Rotterdam, The Netherlands; 2 Department of Internal Medicine, Erasmus MC, Rotterdam, The Netherlands; 3 Department of Immunology, Central University of Ecuador, Quito, Ecuador; 4 Department of Psychiatry, University of Münster, Münster, Germany; 5 Department of Nephrology, Leiden University Medical Center, Leiden, The Netherlands; 6 Prometeo Program SENESCYT, Central University of Ecuador and Universidad de las Fuerzas Armadas, Quito, Ecuador; University of Edinburgh, UNITED KINGDOM

## Abstract

There is increasing evidence that inflammatory macrophages in adipose tissue are involved in insulin resistance of type 2 diabetes (T2D). Due to a relative paucity of data on circulating monocytes in T2D, it is unclear whether the inflammatory changes of adipose tissue macrophages are reflected in these easily accessible cells.

**Objective:**

To study the expression pattern of microRNAs and mRNAs related to inflammation in T2D monocytes.

**Design:**

A microRNA finding study on monocytes of T2D patients and controls using array profiling was followed by a quantitative Real Time PCR (qPCR) study on monocytes of an Ecuadorian validation cohort testing the top over/under-expressed microRNAs. In addition, monocytes of the validation cohort were tested for 24 inflammation-related mRNAs and 2 microRNAs previously found deregulated in (auto)-inflammatory monocytes.

**Results:**

In the finding study, 142 significantly differentially expressed microRNAs were identified, 15 having the strongest power to discriminate T2D patients from controls (sensitivity 66%, specificity 90%). However, differences in expression of these microRNAs between patients and controls were small. On the basis of >1.4 or <0.6-fold change expression 5 microRNAs were selected for further validation. One microRNA (miR-34c-5p) was validated as significantly over-expressed in T2D monocytes. In addition, we found over expression of 3 mRNAs (CD9, DHRS3 and PTPN7) in the validation cohort. These mRNAs are important for cell morphology, adhesion, shape change, and cell differentiation. Classical inflammatory genes (e.g. TNFAIP3) were only over-expressed in monocytes of patients with normal serum lipids. Remarkably, in dyslipidemia, there was a reduction in the expression of inflammatory genes (e.g. ATF3, DUSP2 and PTGS2).

**Conclusions:**

The expression profile of microRNAs/mRNAs in monocytes of T2D patients indicates an altered adhesion, differentiation, and shape change potential. Monocyte inflammatory activation was only found in patients with normal serum lipids. Abnormal lipid values coincided with a reduced monocyte inflammatory state.

## Introduction

There is increasing evidence that monocytes, macrophages and related cells are closely involved in the pathogenesis of the metabolic syndrome (MetS) and type 2 diabetes (T2D). Importantly, in obesity the number of macrophages increases from 10–15% to 50–60% of total cells in adipose tissue [1,2]. The increased secretion of leptin and decreased secretion of adiponectin, by metabolically stressed adipocytes in obesity, results amongst others in the accumulation of macrophages in adipose tissue [3,4]. The increase in macrophage number is accompanied by a hyper activation of the cells and leads to a pro-inflammatory state of the macrophages (so-called M1 type or classically activated macrophages). M1 type macrophages in adipose tissue secrete pro-inflammatory cytokines (TNF-α, IL-1β, IL-6, CCL-4) and chemokines (CCL2), which spill over in the circulation causing a chronic low-grade inflammation [1,3,5–8]. The pro-inflammatory cytokines and chemokines play an important causative role in the insulin resistance of T2D [1,9]

Monocytes are bone marrow-derived and considered to be important circulating precursors for the macrophages in adipose tissue [10–12]. There is however a relative paucity in reports on the state of activation of circulating monocytes in patients with the MetS [13,14] and T2D [15]. In general this state of activation is thought to be pro-inflammatory and increases in pattern recognition receptors (TLRs, NOD-like receptors), oxidative stress and the machinery for the production of pro-inflammatory cytokines have been described [16,17]. The combination of dyslipidemia and chronic hyperglycemia is thought to play a role in this inflammatory activation of the circulating monocytes in MetS and T2D [18,19]. The view has been expressed that further studies on monocyte biology are needed to define the pathogenic role of monocytes/macrophages in MetS and T2D, given that this circulating population is easily accessible and that further clarification of the inflammatory pathophysiology of T2D is needed [20].

Previously, our group carried out such studies on monocyte biology in Latent Onset Diabetes of the Adult (LADA), T2D and Type 1 diabetes (T1D) and reported that diabetic patients exhibit abnormal monocyte gene expression profiles when monocytes were tested for 24 inflammation-related genes (these genes had been detected in earlier carried out gene profiling studies [21]). Two mutually correlating sets of genes (clusters) were found abnormally expressed in the monocytes. A first set (cluster 1) consisted of 12 inflammatory cytokine/compound genes (IL-1B, IL-6, TNF, TNFAIP3, PGS2, CCL20, PTX3, PDE4B, DUSP2, ATF3, CXCL2 and BCL2A1), while a second set (cluster 2) consisted of 12 genes mainly involved in cell motility, chemotaxis, adhesion, differentiation and metabolism (CCL2, CCL7, MAPK6, NAB2, CD9, STX1A, EMP-1, CDC42, PTPN7, DHRS3, FABP5, HSPA1A). Both gene clusters were up-regulated in monocytes of LADA and T2D patients; in juvenile T1D patients only cluster 2 genes were up regulated [21]. Up-regulations of cluster 1 and 2 genes have also been found in the monocytes of other (auto)-inflammatory conditions, such as autoimmune thyroiditis [22] and major mood disorders [23].

Gene expression is partly regulated by a newly discovered level of control, the microRNA system. There is extensive literature indicating that two microRNAs, i.e. miR-146a and miR-155, are key regulators of inflammatory processes [24–32]. An altered expression of these microRNAs has been described in monocytes/macrophages during inflammatory and autoimmune conditions [33–37]. Dysregulation of these microRNAs in peripheral blood mononuclear cells (PBMC) has also been implicated in diabetes [38–40], i.e. miR-146a and miR-155 expression levels were found to be significantly decreased in the PBMCs of patients with T2D compared to control subjects. Moreover, expression values correlated negatively with parameters of metabolic control (Hb1Ac, glucose) and signs of inflammation (NFκB mRNA levels in PBMC, circulatory levels of pro-inflammatory cytokines) [39].

Here we firstly report on a search for abnormally expressed microRNAs in purified monocytes of a cohort of 34 German/Ecuadorian T2D patients using Exiqon array profiling. We tested the found 142 significantly differently expressed microRNAs for discriminating power between patients and non-diabetic controls. Thereafter we selected from the 142 microRNAs 5 microRNAs which were the highest over- or under-expressed with fold changes >1.4x or <0.6x as compared to the non-diabetic controls. We took these 5 microRNAs (miR-138; miR-34c-5p; miR-410; miR-574-3p and miR-576-3p) plus miR-146a and miR-155 for further TaqMan qPCR monocyte studies in a validation cohort of 64 Ecuadorian T2D patients and 44 non-diabetic Ecuadorian controls. In the monocytes of this validation cohort we also tested via qPCR the expression level of the 12 cluster 1 and 12 cluster 2 genes of the earlier reported monocyte inflammatory signature. We finally correlated the expression levels of the tested microRNAs and genes in the validation study to each other (cluster analysis) and to clinical variables, such as age, gender, Hb1Ac and dyslipidemia and studied their discriminative power in distinguishing between patients and controls.

## Materials and Methods

### Subjects for microRNA expression profiling (finding) cohort

Thirty-four subjects diagnosed with type 2 diabetes (T2D), according to the criteria of The Expert Committee on the diagnosis and classification of Diabetes Mellitus [41], were recruited in the German Diabetes Center, Düsseldorf, Germany (Dr Nanette Schloot, *n* = 10) and from three medical centers in Quito, Ecuador (Eugenio Espejo Hospital, Club de Leones Sur, and Fundación de la Psoriasis; *n* = 24 subjects) in 2009. A total of 25 healthy controls (*n* = 9 from Germany and *n* = 16 from Ecuador) with similar ethnic and social background, neither suffering from T2D, nor from other medical disorders (including acute infection), were recruited as well. Studies were performed with approval of the local ethical committees and after informed consent.

### Subjects for the qPCR validation studies

The validation was performed using a new cohort of 64 subjects diagnosed with type 2 diabetes (T2D), according to the criteria of The Expert Committee on the diagnosis and classification of Diabetes Mellitus [41]. Patients were recruited in 4 medical centers of Quito, Ecuador (Eugenio Espejo Hospital, Club de Leones Sur, Fundación Oftalmológica del Valle and Fundación de la Psoriasis) from 2009 until 2012. For demographic and clinical details see Table 1. At the same time, 44 healthy controls with similar ethnical and social background, neither suffering from T2D nor other important medical disorders (including acute infection) served as controls. Controls had to be over 30 years of age (considering the age dependency of T2D). For practical reasons, not in all instances all cases and controls could be tested in the qPCR studies. Exact numbers of tested individuals are indicated.

**Table 1:** Demographic details and clinical characteristics of the validation cohort of Ecuadorian T2D patients and controls.

	T2D		NDC		T2D vs. NDC
					p- Value
Group size n	64		44		
Age mean (range)	61 (37–85)		53 (32–87)		**0.00** **
BMI mean (range) %	29.5 (22–49)	Normal 16.1%	28.7 (23–42)	Normal 18.2%	0.405
		Overweight 40.3%		Overweight 45.5%	
		Obese 43.5%		Obese 36.4%	
**Gender**					
Female n (%)	40 (62.5%)		31 (70.5%)		NA
Male n (%)	24 (37.5%)		13 (29.5%)		NA
**Glucose state**					
Fasting Glucose mg/dL	146 (69–397)	Normal 45.3%	88 (60.9–180.5)	Normal 88.6%	**0.00** **
mean (range) %		High 54.7%		High 11.4%	
HbA1C	7.0 (3.2–12.5)	Normal 35.7%	5.6 (3.9–6.9)	Normal 81.8%	**0.00** **
mean (range) %		High 62.5%		High 18.25%	
**Lipid Profile**					
Cholesterol mg/dL	237 (143–465)	Normal 37.5%	237 (131–328)	Normal 31.8%	0.99
mean (range) %		High 62.5%		High 68.2%	
TG mean mg/dL	205 (76–628)	Normal 60.9%	194 (85–547)	Normal 63.6%	0.56
mean (range) %		High 39.1%		High 36.4%	
HDL mean mg/dL	43 (17–85)	Normal 57.8%	43 (27–87)	Normal 54.5%	0.81
mean (range) %		Low 42.2%		Low 45.5%	
LDL mg/dL	158 (77–395)	Normal 56.3%	158 (78–266)	Normal 50%	0.95
mean (range) %		High 43.8%		High 50%	
**Hepatic Profile**					
ASAT mean mg/dL	33.3 (6.0–78)	Normal 70.8%	41.3 (19–95)	Normal 48.7%	**0.01** *
mean (range) %		High 29.2%		High 51.3%	
ALAT mean mg/dL	38.8 (7.0–131)	Normal 64.6%	44.7 (10–135)	Normal 47.4%	0.252
mean (range) %		High 35.4%		High 52.6%	
**Medication**					
Oral Anti-diabetics	70%		0%		
Insulin treatment	30%		0%		
Statins (%)	0%		0%		

Values in bold denote a significant difference between two groups.

*p0.01,

**p0.001.

Table 1 shows sample sizes, distributions of age, gender, comorbidities, HbA1c/hyperglycemia, BMI, hepatic profile, lipid profile, and medication use of the patient and control groups.

In all cohorts, patients and healthy controls with other immune disorders, other serious medical illnesses, recent infections (last 2 weeks), obvious vascular complications such as diabetic foot and ulcers, fever, pregnancy/postpartum and LADA patients (patients positive for GAD-65 Abs) were excluded. None of the patients used statins. The Medical Ethical Review Committee of the Ecuadorian Corporation of Biotechnology Quito, Ecuador and the Ethic Committee of the Central University of Quito approved the study. Written informed consent was obtained of all subjects participating in the study. The Ecuadorian Ministry of Health (MSP) gave the permit to export and process the samples in Erasmus MC, Rotterdam, The Netherlands.

### Blood collection and preparation

Blood (drawn in the morning) was collected in tubes containing sodium-heparin for immune cell preparation. From the heparinized blood, peripheral blood mononuclear cell (PBMC) suspensions were prepared in the afternoon by low-density gradient centrifugation, as previously described in detail [42] within 8 h to avoid activation of the monocytes. PBMCs were frozen in 10%-dimethylsulfoxide and stored in liquid nitrogen. This enabled us to test patient and control immune cells in the same series of experiments later.

### Isolation of monocytes

CD14-positive (CD14^+^) monocytes were isolated from thawed PBMCs by a magnetic cell sorting system (MACS; Miltenyi Biotec, Auburn, California). The purity of monocytes was >95% (determined by morphological screening after Trypan Blue staining and flow cytometric analysis). As previously reported; the positive vs. negative selection of immune cells did not influence gene expression profiles [43].

### MicroRNA microarray hybridization

Total RNA was extracted from purified monocytes using a mirVana miRNA isolation kit (Ambion) according to the manufacturer’s protocols. RNA was labeled using a ULS RNA labeling kit (KreatechDiagnostics, Amsterdam). To that end, 1.5 μg of total RNA was incubated with Cy3-ULS for 30 min at 85°C and purified to remove unbound Cy3-ULS. Labeled RNA was hybridized on miRCURY LNA microRNA arrays (probe set 10.0; Exiqon, Vedbaek, Denmark) at 60°C for 16h using a Tecan 4800 hybridization station. Slides were washed and immediately scanned using a Tecan LSRe loaded microarray laser scanner.

### microRNA RT qPCR assays

Total RNA was isolated from purified monocytes using the mirVana miRNA Isolation Kit (Ambion) as described by the manufacturer’s manual. Purity and integrity of the RNA were assessed on the Agilent 2100 bioanalyzer with the RNA 6000 Nano LabChip reagent set (Agilent Technologies, Santa Clara, CA, USA) and the RNA was then stored at −80°C. Subsequently, specific stem-looped reverse transcription primers were used to obtain cDNA for mature microRNAs. The RNA was reverse transcribed using the TaqMan MicroRNA Reverse Transcription Kit from Applied Biosystems, The Netherlands (ABI). PCR was performed using pre-designed TaqMan microRNA assays and TaqMan Universal Master Mix, NoAmpEraseUNG, with an ABI 7900 HT real-time PCR machine. The PCR conditions were 2 min at 50°C, 10 min at 95°C, followed by 40 cycles of 15s at 95°C, and 1 min at 60°C.

### mRNA expression analysis in monocytes via TaqMan Array Cards

One μg of RNA was reverse-transcribed using the High Capacity cDNA kit (Applied Biosystems, Foster City, CA, USA). qPCR was performed using custom TaqMan Arrays, format 48 (Applied Biosystems), according to the manufacturer’s protocol and validated against the single RT-qPCR method. Per fill port, 400 ng of cDNA (converted from total RNA) was loaded. PCR amplification was performed using an Applied Biosystems Prism 7900HT sequence detection system with TaqMan Array block. Thermal cycler conditions were 2 min at 50°C, 10 min at 94.5°C, and then 30s at 97°C, and 1 min at 59.7°C for 40 cycles. Relative to the housekeeping gene ABL1, the expressions of ATF3, BCL2A1, CCL20, CCL2, CCL4, CD9, CDC42, CXCL2, DHRS3, DUSP2, EMP1, FABP5, HSPA1A/HSPA1B, IL-1B, IL-6, MAPK6, NAB2, PDE4B, PTGS2, PTPN7, PTX3, STX1A, TNF, and TNFAIP3 were determined and values were calculated using the comparative threshold cycle (Ct) method. ABL was chosen as a reference gene because it was previously shown that ABL was the most consistently expressed reference gene in hematopoietic cells [44]. The quantitative value obtained from qPCR is a cycle threshold (Ct). The fold change values between different groups were determined from normalized Ct values (Ct gene—Ct housekeeping gene), by the ΔΔCt method.

### Data analysis microRNA microarray

Microarray data extraction and normalization was carried out as described previously [45]. We analyzed 711 microRNAs using Empirical Bayesian method for assessing differential expression (R package limma) to detect microRNAs differentially expressed between cases and controls. For outlier detection, we used Grubb's test for individual microRNA (threshold for significance 0.05). Outliers were replaced by a median expression value. The Benjamin-Hochberg method (5% false discovery rate) was applied to correct for multiple testing. Target genes of the identified microRNAs were predicted using miRecords (http://www.mirecords.bioled.org). Functional annotation of the predicted genes was performed using Ingenuity Pathway Analysis (Ingenuity Systems).

### Data analysis RT qPCR

The SDS software (ABI) was used to collect the data and the RQ Manager Program (ABI) was used to assign, check, and standardize Ct values. Data Assist software (ABI) was used to normalize the data to ABL for mRNA expression and RNU44 for microRNA expression. For threshold cycles below 40, the corresponding microRNA was considered detected, higher cycle numbers were not included in calculations. The results were quantified using the ΔΔCt method (2−ΔΔCt, User Bulletin 2, ABI). Statistical analysis was performed using the SPSS (IBM, Inc.) package for Windows. Data were tested for normal distribution using the Kolmogorov-Smirnov test. The Grubbs' test for outlier detection was applied (http://graphpad.com/support/faqid/1598/). Depending on the distribution pattern and the total number of subjects, parametric (normal distribution, independent t test) or nonparametric group comparison (Mann-Whitney U test) were applied. Correlations were determined by Spearman’s correlation coefficient. Levels of significance were set at p ≤ 0.05 (two tailed). A dendrogram visualizing associations was constructed in SPSS using hierarchical cluster analysis of the genes and microRNA expression using the between-groups linkage method. Graphs were designed with Illustrator CS6 for Windows.

## Results

### Exploratory search for T2D-related monocyte micro-RNAs using Exiqon arrays

To investigate T2D-related monocyte microRNA profiles, we profiled the monocytes of 34 T2D patients (age: 22–77 years, mean 55 years) and of 25 non-diabetic controls (age: 31–71 years, mean 49 years) of the finding cohort. After correction for multiple testing (Benjamin-Hochberg method), we detected 142 microRNA differentially expressed in T2D patients compared to controls. From the 142 microRNAs, 49 microRNAs (35%) were down-regulated and 93 microRNAs (65%) were up-regulated. The list is available in the supporting information files of this article (S1 Table). Using Ingenuity pathway analysis with inclusion of only literature-confirmed targets of the identified microRNAs, we found that SOCS4 and SOCS6 genes ranked highest as potential targets of these differentially expressed microRNAs in monocytes, suggesting that especially inflammatory networks were regulated by these microRNAs.

Additionally, computational class prediction analysis was performed with the 142 significantly different expressed microRNAs using the LASSO model of penalized prediction. This showed that 15 microRNAs indicated an optimal prediction signature (underlined in S1 Table). Using the data on expression of these microRNAs as determined in array, we clustered patients and controls of the finding cohort by unsupervised hierarchical clustering (S1 Fig). Indeed, this approach showed that subject clusters can be identified with a first cluster containing 24 T2D cases and only 2 healthy controls, and a second mixed cluster comprising 12 cases and 23 healthy controls (sensitivity 66%, specificity 90%). Thus, using microRNAs, we found that a partial separation can be made between T2D cases and controls. These prediction signature microRNAs, however, appeared less useful to validate as microRNAs that can be used as discriminating parameters between T2D patients and controls in a separate cohort using qPCR as an independent technique, since the expression fold changes observed for these microRNAs were generally too low to allow reliable confirmation within the technical limitations of qPCR. Therefore, we chose to select from the differentially expressed microRNAs those with the highest fold changes (FC) between cases and controls with FC of >1.4 or <0.6. Another criterion for selection was that TaqMan probes and primers needed to be available.

### Validation studies of 5 selected T2D-related monocyte microRNAs using qPCR

From the 142 differentially expressed microRNAs found in Exiqon, 5 microRNAs fulfilled the selection criteria: miR-138; miR-34c-5p; miR-410; miR-574-3p and miR-576-3p. Additionally, we tested microRNAs-146a and -155 in TaqMan analyses, since these microRNAs are well-known regulators of inflammation, and have been identified in T2D PBMC by others [38,39].

**Fig 1:**
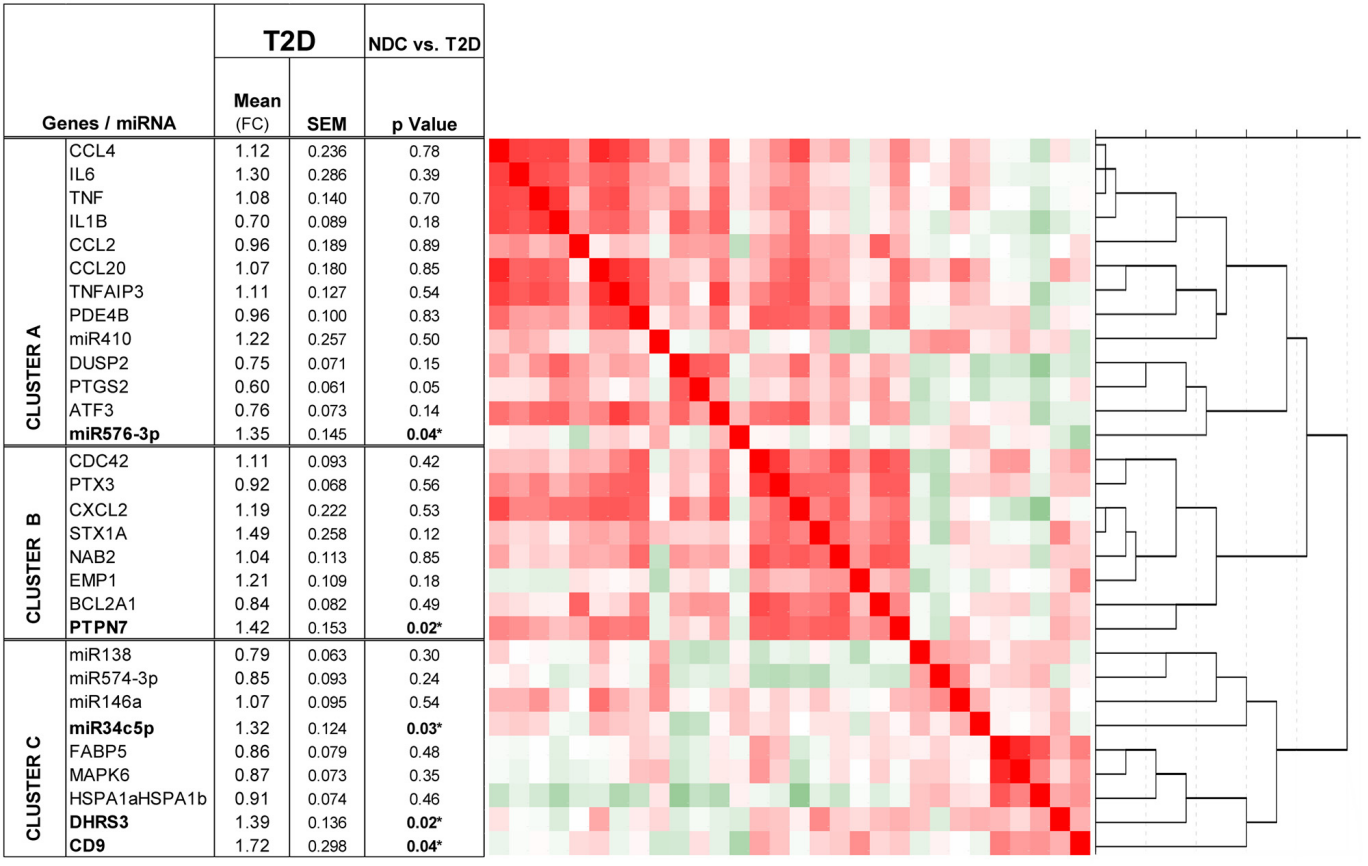
Hierarchical cluster analysis of the tested genes and microRNAs of the monocytes of Ecuadorian type 2 diabetic patients and controls in the validation cohort. On the left, the fold change values between the T2D group and the non-diabetic controls were determined from normalized Ct values (Ct gene/Ct reference gene ABL) by the ΔΔCt method (2−ΔΔCt, User Bulletin 2; Applied Biosystems, Foster City, CA). Data were standardized to the non-diabetic control subjects. The fold change of each gene in the non-diabetic control subjects is therefore 1. Differences between groups were tested using t tests for independent samples. This table shows that 2 microRNAs (MiR-34c-5p and miR-576-3p) were significantly higher expressed in the monocytes of the T2D patients compared to non-diabetic controls. Also, 4 genes (of the 24 tested) were significantly different expressed (PTGS2 lower, and CD9, DHRS3 and PTPN7 significantly higher). The heatmap and dendrogram present the result of the hierarchical clustering of the genes. Three major clusters were found: Cluster A contains inflammatory compounds and includes miR-410 and miR-576-3p. Cluster B contains inflammatory compounds and factors involved with migration/differentiation/metabolism; Cluster C only consists of migration/metabolic factors. MiR-138, miR-574-3p, miR-146a and miR-34c-5p formed a sub-cluster within cluster C and strongly clustered together.

The table of Fig 1 shows (apart from other data) the expression level of the 5 microRNAs in the monocytes of the Ecuadorian validation cohort of type 2 diabetic patients and non-diabetic controls (in total 48 patients and 34 controls could be used). Of the microRNAs, miR-155 was not or hardly detected in the monocytes using TaqMan qPCR array techniques, and therefore data are not given. The table of Fig 1 shows that of the tested microRNAs, MiR-34c-5p and miR-576-3p were significantly higher expressed in the monocytes of the type 2 diabetic patients as compared to the monocytes of the non-diabetic controls.

Since our patients and controls of the validation cohort differed on average 8 years in age, we took special notice of correlations of molecular parameters with age. MiR-576-3p correlated significantly and positively with age (r = .257; p = .02), whereas miR-34c-5p did not (r = .041; p = .713). It is important to note that correction for age resulted in loss of significance in the association of T2D with the expression of miR-576-3p. We therefore consider higher expression of this microRNA as related to age rather than to disease. We thereafter performed a correlation analysis between the level of miR-34c-5p and dyslipidemia, hyperglycemia and liver function. This analysis showed that the expression level of miR-34c-5p was not determined by these factors but was only associated with T2D.

### Target prediction of miR-34c-5p

Since the expression of miR-34c-5p was significantly up-regulated in T2D monocytes, we asked if there were *in silico* indications linking miR-34c-5p expression to the regulation of inflammation. We used miRecords as a resource for microRNA-target interactions as this web-based tool integrates predicted microRNA targets produced by 11 established microRNA target prediction programs (DIANA-microT, MicroInspector, miRanda, MirTarget2, miTarget, NBmiRTar, PicTar, PITA, RNA22, RNAhybrid and TargetScan/TargetScanS, available at http://www.mirecords.bioled.org).

Minimum target gene prediction coverage of three algorithms was used to perform prediction analysis for miR-34c-5p, which resulted in 4291 hits.

Ingenuity pathway analysis (Ingenuity Systems) was used for mapping of the predicted target genes to biological functions. Interestingly, the top molecular and cellular function of the miR-34c-5p predicted target genes was “cell morphology” (S1 Text); while the second top-associated network was “cell morphology/cellular assembly and organization/cellular development”. In the top canonical pathways, the STAT3 pathway was third in line.

Among the potential targets of miR-34c-5p, some of the diabetes-related signature genes identified earlier [21] were found, i.e. PTGS2, PDE4B and EMP1 were predicted as targets of miR-34c-5p in three to five algorithms. Interestingly, all our predicted targets derived from the same 6 (miRanda, Mir Target2, PicTar, PITA, RNAhybrid and Target Scan) of the 11 algorithms integrated by miRecords.

### TaqMan qPCR analysis for the expression of the 24 signature mRNAs in the monocytes of the Ecuadorian validation cohort

We carried out a qPCR analysis for the expression of the 24 cluster 1 and 2 mRNAs using the monocytes of the validation cohort of the Ecuadorian T2D patients and controls. The levels of the gene data are expressed in the table of Fig 1 (which also shows the cluster analysis of the genes and the tested microRNAs, see for explanation underneath). The table shows that of the 24 genes tested, 4 genes were significantly differentially expressed in the monocytes of the T2D patients as compared to the monocytes of the non-diabetic controls (in total material of 43 patients and 33 controls was available).

PTGS2 was significantly lower expressed in the T2D monocytes, while CD9, DHRS3 and PTPN7 were significantly higher expressed in the monocytes of the T2D patients as compared to the non-diabetic controls.

Since our patients and controls of the validation cohort differed on average 8 years in age, we again took special notice of correlations of the expression of these genes with age. There were no age- or gender-dependencies of these 4 abnormally expressed genes. We also performed correlation analyses with hyperglycemia, dyslipidemia, and liver function, but did not find statistically significant correlations.

### Interdependence of microRNA and mRNA expression in T2D monocytes

To study the mutually inter-dependent state of mRNAs and microRNAs in expression, we also performed a cluster analysis on the qPCR data of both the mRNAs and microRNAs. The dendrogram of this analysis is also given in Fig 1. Three main clusters of mutually correlating genes and microRNAs could be identified; we arbitrarily called these clusters A, B and C.

Cluster A consisted predominantly of genes originally found in Padmos et al. [21] on diabetic monocytes to belong to the cluster of inflammatory genes (previously called “cluster 1” in Padmos et al, 23). These genes are well-known inflammatory compound genes, such as genes for the pro-inflammatory cytokines IL1B, IL6 and TNF, the inflammatory compound PTGS2/COX2, inflammatory chemokines (CCL20, CCL2, CCL4) and transcription factors and regulators of inflammatory pathways such as PDE4B, DUSP2 and ATF3 (Fig 1). Also miR-410 and the age-related miR-576-3p appeared to be part of this inflammatory cluster.

Cluster B also consisted of genes earlier found in the cluster of inflammatory genes (“cluster 1”), such as CXCL2, PTX3 and BCL2A1. However, this cluster also contained genes originally found by Padmos et al. to belong to the cluster of adhesion/motility/differentiation/metabolic factors (“cluster 2”) [21], such as CDC42, STX1A, NAB2, EMP1 and PTPN7.

A third cluster C consisted exclusively of genes found earlier in the cluster of adhesion/motility/differentiation/metabolic factors of Padmos et al (“cluster 2”), such as CD9, DHRS3, FABP5, MAPK6 and HSPA1. Interestingly, miR-138, miR-34c-5p, miR-146a and miR-574-3p formed a sub-cluster within cluster C and strongly clustered together.

### Clustering of the diabetic patients and non-diabetic controls of the validation cohort using microRNA and mRNA expression

**Fig 2:**
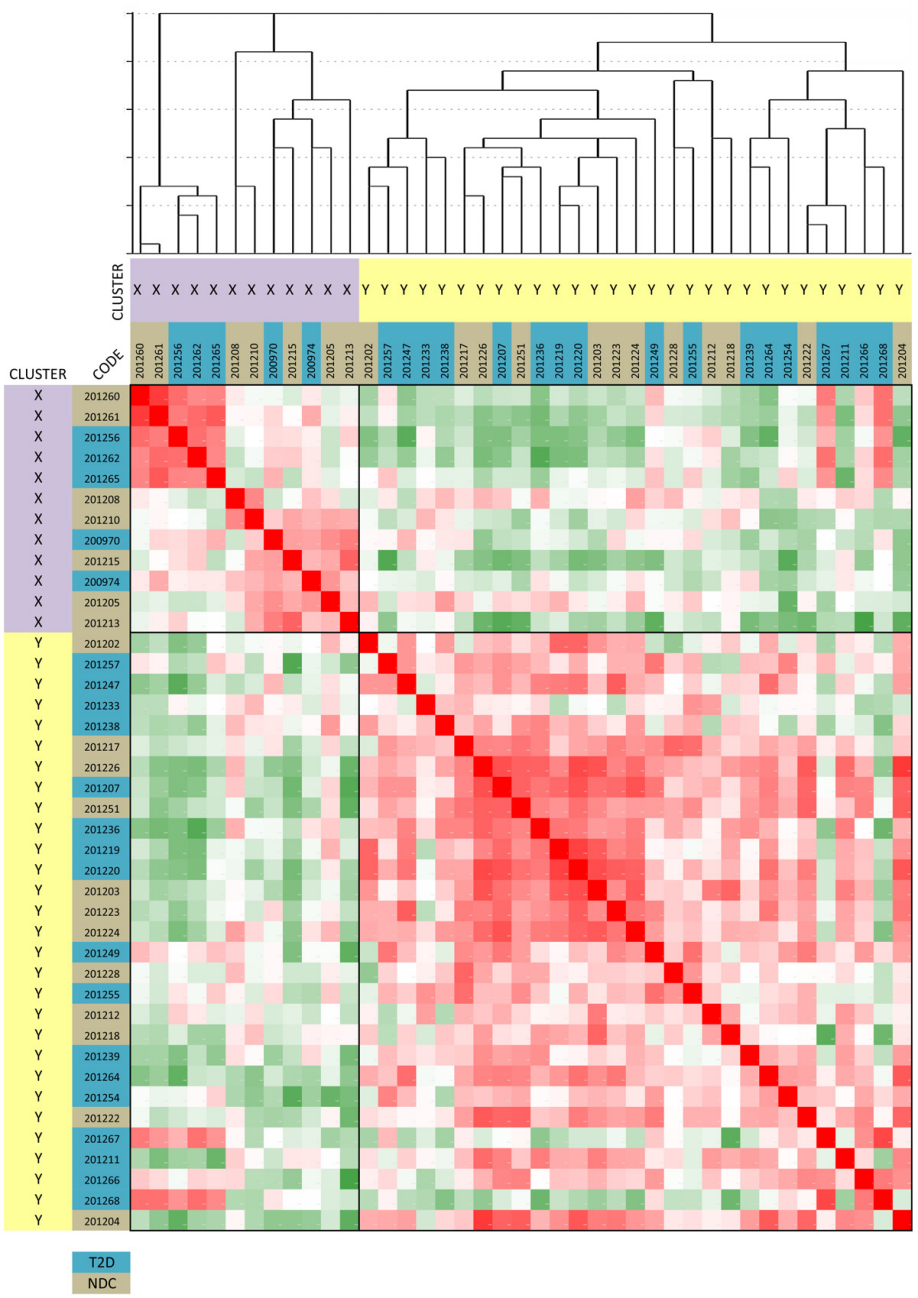
Dendrogram and heatmap of hierarchical clustering of T2D patients and non-diabetic controls of the validation cohort using microRNA and mRNA expression as determined by qPCR. This figure shows that two main subject clusters (X and Y) could be identified. Cluster X contained 5 diabetics and 7 non-diabetic subjects, and cluster Y comprised 17 diabetics and 12 non-diabetics. This approach did not distinguish between T2D patients and non-diabetic controls.

Using the data on expression of the mRNAs and microRNAs of the validation study, we also clustered diabetics and non-diabetics by unsupervised hierarchical clustering (Fig 2) (in 22 patients and 19 controls both mRNA and microRNA studies had been performed). This approach showed that two main subject clusters were identified with a first cluster (cluster X) containing 5 diabetics and 7 non-diabetic subjects, and a second cluster (cluster Y) comprising 17 diabetics and 12 non-diabetics, showing that a clinically useful distinction between diabetes and non-diabetes could not be made using the selected mRNAs and microRNAs.

However, it also emerged from the data that the subject clustering made a distinction on the basis of dyslipidemia, particularly in the diabetic group (see Table 2): The diabetics in the cluster X had virtually normal levels of cholesterol, LDL and triglycerides, while the diabetics of cluster Y had significantly higher levels of cholesterol, LDL and triglycerides. With regard to monocyte gene expression cluster X of diabetics with normal lipid values had pro-inflammatory monocytes, with up-regulation of many cluster A genes, which reached significance for TNFAIP3 (HSPA1 of gene cluster C was down-regulated). Remarkably, the second cluster of diabetics (Y) with high lipid values had reduced expression of most of these pro-inflammatory genes, reaching significance for DUSP2, ATF3 and PTGS2. MicroRNAs were not significantly differentially expressed in the groups (except for perhaps miR-138, which tended to be lower in the diabetics with normal lipids).

**Table 2:** Distinction of non-diabetic controls and T2D patients on the basis of dyslipidemia.

Genes/ miRNA		T2D Cl-X		T2D Cl-Y			NDC Cl-X	NDC Cl-Y	
		Mean (FC)	p value vs N-T2D	Mean (FC)	p value vs N-T2D	p value CLX vs CLY	Mean (FC)	Mean (FC)	p value CLX vs CLY
**CLUSTER A**	**CCL4**	3.76	0.19	0.85	0.44	0.12	2.96	0.36	0.11
	**IL6**	4.49	0.11	0.87	0.37	0.09	2.13	0.63	**0.04**
	**TNF**	2.47	0.21	0.93	0.45	0.15	1.75	0.81	0.21
	**IL1B**	1.74	0.28	0.60	0.07	**0.02**	2.58	0.42	**0.01**
	**CCL2**	3.08	0.17	0.64	0.13	0.10	1.64	0.92	0.43
	**CCL20**	1.69	0.19	0.80	0.62	**0.04**	2.42	0.24	**0.05**
	**TNFAIP3**	**2.06**	0.05	1.05	0.88	**0.05**	1.71	0.73	0.10
	**PDE4B**	1.33	0.28	0.90	0.72	0.21	1.52	0.67	**0.02**
	**miR410**	1.59	0.66	1.79	0.30	0.88	0.99	1.03	0.96
	**DUSP2**	1.21	0.79	**0.56**	**0.03**	0.06	2.07	0.56	**0.00**
	**PTGS2**	0.82	0.41	**0.50**	**0.05**	0.28	2.09	0.61	0.08
	**ATF3**	1.50	0.33	**0.66**	**0.04**	**0.03**	1.68	0.85	0.14
	**miR576-3p**	1.29	0.42	1.03	0.81	0.46	0.75	1.14	**0.04**
**CLUSTER B**	**CDC42**	1.44	0.13	0.96	0.80	0.10	1.14	0.92	0.53
	**PTX3**	1.84	0.15	1.08	0.13	**0.03**	1.62	0.82	0.12
	**CXCL2**	3.55	0.19	0.71	0.17	0.13	2.38	0.55	0.06
	**STX1A**	2.98	0.34	0.89	0.62	0.31	1.66	0.81	0.12
	**NAB2**	1.32	0.70	0.74	0.25	0.26	1.83	0.68	0.19
	**EMP1**	1.64	0.52	1.12	0.92	0.53	1.23	0.99	0.63
	**BCL2A1**	1.07	0.87	0.72	0.28	0.13	1.64	0.83	0.42
	**PTPN7**	1.84	0.08	1.08	0.77	0.12	1.44	0.74	0.07
**CLUSTER C**	**miR138**	0.58	0.06	1.04	0.87	0.07	0.97	1.10	0.62
	**miR574-3p**	0.72	0.16	0.87	0.19	0.40	0.77	1.14	**0.05**
	**miR146a**	1.10	0.56	1.00	0.89	0.64	0.79	1.10	0.09
	**miR34c5p**	1.28	0.08	1.16	0.19	0.39	0.91	1.07	0.17
	**MAPK6**	0.58	0.07	1.03	0.93	0.07	1.08	0.96	0.70
	**HSPA1aHSPA1b**	**0.49**	**0.04**	0.99	0.99	**0.04**	0.91	1.04	0.42
	**DHRS3**	1.11	0.23	1.31	0.11	0.55	0.72	0.88	0.37
	**CD9**	1.32	0.54	1.93	0.13	0.37	0.67	1.22	0.24
**Cholesterol**		**202**		235		**0.04**	231	258	0.23
**LDL**		**119**		177		**0.03**	151	179	0.22
**Triglycerides**		**151**		238		**0.03**	220	182	0.55

Table 2 shows that the T2D patients in the cluster X had virtually normal levels of cholesterol, LDL and triglycerides. The T2D patients of cluster Y had significantly higher levels of cholesterol, LDL and triglycerides. The monocyte gene expression of cluster X (T2D with normal lipid values) had up-regulation of many pro-inflammatory genes (cluster A), which reached significance for TNFAIP3. The monocyte gene expression of cluster Y (T2D with high lipid values) had reduced expression of most of the pro-inflammatory genes (cluster A), reaching significance for DUSP2, ATF3 and PTGS2. MicroRNAs were not significantly differentially expressed in the groups. In the HC subjects, this type of clustering also made a distinction in cluster X (HC with high expression of inflammatory genes) and cluster Y (HC with reduced expression of inflammatory genes). Although the HC group also had higher cholesterol and LDL levels these did not reach statistical significance.

In the non-diabetic subjects, this type of clustering also made a distinction in cluster X subjects with a high expression of gene cluster A inflammatory genes and cluster Y subjects with a reduced expression of these genes. Although the latter non-diabetic subject group also had higher cholesterol and LDL levels these did not reach statistical significance.

## Discussion

In this study, we found 15 discriminating microRNAs in a screening study on the purified monocytes of T2D patients, a distinction could be made between T2D patients and non-diabetic controls with a sensitivity of 66% and specificity 90%. Although the specificity is acceptable, sensitivity is relatively low. Moreover, the 15 discriminating microRNAs were only marginally lower and higher expressed. We therefore chose to continue our search and to validate only discriminating microRNAs with a significant fold change of 1,4 or 0,6 versus non-diabetics in a qPCR study using the monocytes of a new series of Ecuadorian patients, of whom we had detailed clinical information.

Of the selected 5 microRNAs only miR-34c-5p was validated as significantly higher expressed in the circulating monocytes of the validation cohort of T2D patients, also taking confounding factors as age, gender, BMI, liver function and dyslipidemia into consideration.

With regard to previous literature on the validated expression of microRNAs in peripheral blood leukocytes in T2D, there are 5 relevant reports [38,39,46–48]. In none of these reports purified monocytes have been tested. In total 9 microRNAs were found abnormally expressed in these reports and interestingly miR-34a, which has virtually the same sequence and targets as miR-34c-5p, was found to be down-regulated in T2D patients upon resveratrol treatment [47].

In Ingenuity analysis the top molecular and cellular function of miR-34c-5p predicted target genes was “cell morphology”. With regard to the literature on the role of miR-34c-5p in cell biology there are only few reports. The scarce literature shows a role of this microRNA in diverse cellular processes, such as inflammatory responses [49]; growth, apoptosis [50] [51], invasiveness of tumor cells [40], and cell morphological processes involved in the differentiation and the morphogenesis of neuronal cell projections [52].

Interestingly, a recent report shows that miR-34c-5p has a function in altering the expression of c-Met, the receptor for HGF, also known as scatter factor [53]. HGF is amongst others an angiogenic factor and plays a role in endothelial migration, proliferation and neovascularization. Also, we recently carried out a preliminary study in which we profiled T2D monocytes for microRNA expression in relation to their capacity to form pro-angiogenic cells. Pro-angiogenic cells are also known as myeloid endothelial progenitor cells and play a role in vascular repair [54]. When we compared the group of high pro-angiogenic cell formers with those with a low potential for pro-angiogenic cell formation, miR-34c appeared to be the most discriminating microRNA, being raised 10 times in the high pro-angiogenic cell formers (data to be published). With regard to the literature on pro-angiogenic cells others have found microRNAs miR-126, miR-130, miR-21, miR-27) to be lower expressed in pro-angiogenic cells of T2D patients [55]. Collectively our *in silico* targeting data, the literature data and our preliminary data on pro-angiogenic cells supports a view that the raised expression of miR-34c-5p in T2D monocytes might represent a molecular sign for a raised potential of the T2D monocytes for cell morphological changes and differentiation to vascular support cells to compensate increased endothelial damage in T2D.

This concept is also supported by the mRNA expression in the monocytes of the Ecuadorian validation cohort: We found a significant up-regulation of CD9, DHRS3 and PTPN7. These genes were earlier described as up-regulated in monocytes of T2D, LADA and T1D patients by Padmos et al and Beyan et al.[21,56]. CD9 is a tetraspanin and an important regulator of integrin activity and plays a role in the immunological synapse as well as in endothelial adhesion and transmigration [57,58]. DHRS3 is an enzyme involved in vitamin A (retinoid) metabolism and vitamin A is an important growth and differentiation factor for immune cells and their precursors [59–61]. PTPN7 (or HePTP) is a tyrosine phosphatase regulating the activity of p38 and ERK playing an important role in the differentiation of monocytes to progeny cells including macrophages, dendritic cells and pro-angiogenic cells. Moreover, CD9 and DHRS3 clustered in cluster analysis with the expression of miR-34c-5p in monocytes, underscoring a putative role of all these molecules in processes of cell differentiation and cell morphogenesis. Taking these data together, we therefore assume that our observation on up-regulated CD9 and DHRS3 and PTPN7 expression in T2D monocytes can (next to the over-expression of miR-34c-5p) be taken as a sign that the circulating monocytes in T2D patients have an altered potential for adhesion, migration and differentiation into progeny, such as macrophages, dendritic cells and vascular support cells.

This study does not support the view that monocytes are *in general* pro-inflammatory activated in T2D patients: The circulating monocytes of the T2D patients of the validation cohort failed to show a significant up-regulation of typical pro-inflammatory genes (such as IL-1B, IL-6, TNF, CCL4 and CCL20) as compared to monocytes of non-diabetic controls with a similar ethnic background, considering the T2D patient population in total. Some important pro-inflammatory genes were even down-regulated (DUSP2, ATF3) and down-regulation reached significance for PTGS2. Also miR-146a, which is a classical microRNA dampening inflammatory responses and earlier found as down-regulated in monocytes and macrophages of patients with (auto-)inflammatory conditions [38], was not reduced in the monocytes of our T2D Ecuadorian cases as compared to the non-diabetic controls (it was also not identified in the finding cohort). Our current data thus refute the earlier expressed views in literature [15], and by us [21], that circulating monocytes of T2D patients are *in general* characterized by a pro-inflammatory state.

However when we divided the Ecuadorian T2D and non-diabetic subjects on the basis of a subject cluster analysis using the expression data of the 24 mRNAs and 6 microRNAs, we identified two sets of subjects.

The diabetics in the first set were characterized by virtually normal serum lipid values. These patients did have a raised monocyte expression of pro-inflammatory cluster A genes, reaching significance for TNFAIP (A20), an important TNF-induced inflammatory gene.

The patients of the second set of subjects were characterized by raised cholesterol, LDL and triglycerides and a down-regulation of pro-inflammatory monocyte genes, reaching significance for DUSP2, ATF3 and PTGS2 (COX2). DUSP2 and ATF3 are important transcription regulators of inflammatory compounds, while PTGS2 is a well-known enzyme of the prostaglandin pathway.

The patient cluster data thus suggest that pro-inflammatory monocytes do circulate in T2D patients provided there is a normal serum lipid state. In case of dyslipidemia circulating monocytes have a significantly reduced expression of typical pro-inflammatory genes.

With regard to dyslipidemia being associated with reduced expression of pro-inflammatory genes, it is important to note that our Ecuadorian general population control group was atypical in also having many signs of dyslipidemia: Hypercholesterolemia was present in 68% and a raised LDL in 40% (BMIs were over 25 in 83% of the population). Considering the excessively high prevalence of dyslipidemia (and obesity) in the Quito general population control group, it is important to note that a recent healthcare report of the Ecuadorian government corroborates this high prevalence of dyslipidemia and obesity in urban Ecuadorian populations [62]. One can therefore ask the question whether, contrary to expectation, also in the Quito *controls* the high serum lipids might be associated with a down regulation of the inflammatory genes in circulating monocytes.

Our subject cluster analysis on the validation cohort delivered indeed two groups of non-diabetic Quito controls differing in pro-inflammatory state of monocytes; the non-diabetics of subject cluster Y had anti-inflammatory monocytes with various significantly down-regulated cluster A genes. Although their lipid values were higher, they did not reach statistical significance. To further investigate the concept of dyslipidemia being associated with an anti-inflammatory state of circulating monocytes, we thereafter compared in preliminary studies the monocytes of the Quito general population control group with those of a Dutch general population control group, which had been collected and analyzed at the same time as the Ecuadorian controls. The Dutch general population controls had normal lipid values (hypercholesterolemia none, raised LDL 14%), contrasting to the much higher values found in the Ecuadorian general population controls. We used for monocyte gene expression the same type of TLDA qPCR cards. When we compared outcomes of both groups (see S2 Table), we indeed found that the monocytes of the Ecuadorian non-diabetic general population controls with the high rate of abnormal serum lipids had significantly reduced expression levels of many of the classical inflammatory cluster A and B genes as compared to the Dutch general population controls. Cluster C genes were largely unaltered in the monocytes of the group of dyslipidemia Ecuadorian non-diabetic general population controls. These data therefore strengthen the view that dyslipidemia is associated with a dampened inflammatory state of circulating monocytes. However a word of caution is here also in place; the usage of aspirin and NSAIDs is high in the general population of Ecuador [63,64], and drug effects may therefore also have played a role in the difference in monocyte inflammatory state between the Dutch and Ecuadorian controls.

It is further of interest to note that the found raised microRNA miR-34c-5p may have played a mechanistic role in the phenomenon of down regulated inflammatory gene expression in monocytes. *In silico* we found the inflammatory genes PTGS2 and PDE4B as direct targets of miR-34c-5p, and although functional studies are required to formally test such target interactions, these *in silico* data suggest that miR-34c-5p might be instrumental in the down regulation of the inflammatory state of the monocytes.

Remains the question how our abnormal monocyte gene expression relates to the genetic back ground of T2D subjects. Interestingly, genome-wide association studies for T2D have highlighted multiple genes implicated in adipo-cytokine pathways and cell cycle regulation [65,66]. Many of the genes of our monocyte gene cluster A are part of the adipo-cytokine network, further highlighting the etiological relevance of these pathways. However, it is unlikely that any genetic variation would be explaining the here observed gene expression differences in monocytes. All of the known common genetic variants for T2D have too small effect sizes that they would not discriminate between case/control status in this study [66]. Complex gene-environment (such as obesity and dyslipidemia) interactions most likely play a role in the here described aberrant monocyte gene expression. It must also be noted in this report on Ecuadorian patients that, although the T2D related GWAS polymorphisms have largely been detected in populations of white European ancestry, many are also prevalent in other ethnic groups [67].

## Limitations

In retrospect our diabetic and non-diabetic control groups between the finding and validation studies differ considerably with regard to parameters that do influence the microRNA and mRNA profiles, such as lipid values (and use of statins that influence lipid values) and perhaps ethnicity (finding cohort comprised Germans and Ecuadorians, validation cohort only Ecuadorians). On the other hand our studies using these groups have unveiled here a hitherto unknown effect of abnormal lipids on circulating monocytes rendering them most likely better cells for vascular repair. In ongoing studies we make use of Dutch diabetic and non-diabetic subject to investigate whether this negative association between lipid profile and monocyte inflammatory state can be confirmed in Caucasian diabetic populations (next reports).

Furthermore we used for the microRNA studies TaqMan qPCR assays for validation, after having used Exiqon arrays in the finding study. In a limited series we were able to also validate some of the microRNAs with Exiqon qPCR and it turned out that sensitivities for detection differed in some instances between the two methods for validation (Exiqon versus TaqMan qPCR), e.g. miR-155 was not detectable in the monocytes via TaqMan qPCR, while it was readily detected via Exiqon array and qPCR. On the other hand, miR-410 was better detectable by TaqMan qPCR. Therefore follow up studies should preferably include more extensive comparisons between the two detection methods to select for robust discriminating microRNAs.

## Conclusion

Using microRNA and mRNA profiling and validation we found an over-expression of miR-34c-5p and of a set of three genes (CD9, DHRS3 and PTPN7) in the monocytes of Ecuadorian T2D patients suggesting an altered adhesion, differentiation potential and shape change potential of the circulating monocytes. We assume that this increased potential might be instrumental in vascular repair. With regard to inflammatory genes we only found a pro-inflammatory state of monocytes in T2D patients with normal serum lipids (who formed a minority within the diabetic group). Dyslipidemia coincided with a reduced expression of pro-inflammatory genes in circulating monocytes, which might be instrumental in strengthening the potential of monocytes for vascular repair.

## Supporting Information

S1 Fig Dendrogram and heatmap of hierarchical clustering of T2D patients and non-diabetic controls of the finding cohort using microRNAs. This figure shows that partial separation can be made between T2D patients and healthy controls on the basis of the 15 microRNAs identified as optimal prediction signature. Two main subject clusters were identified. The first cluster contain 24 T2D patients (yellow) and only 2 healthy controls (blue), and the second mixed cluster contains 12 patients (yellow) and 23 healthy controls (blue).
(PDF)

S1 Table Differentially expressed monocyte microRNAs of T2D patients compared to non-diabetic controls of the finding cohort. This table shows the 142 microRNAs that were found to be differentially expressed in T2D patients compared to controls. 35% of the miRNAs were down-regulated and 65% were up-regulated.
(DOCX)

S2 Table Monocyte gene expression of the Ecuadorian non-diabetic controls vs. the Dutch non-diabetic controls. Values represent the means and standard deviations of normalized Ct values (Ct gene/Ct reference gene ABL) by the ΔΔCt. Genes are given in the order of the cluster diagram given in this paper. The table shows significantly reduced expression levels of many of the classical inflammatory cluster A and B genes in the Ecuadorian non-diabetic controls compared to the Dutch controls. Cluster C genes were largely unaltered in the monocytes of the Ecuadorian group. Of note, these analyses were performed in the same time period to exclude technical variability.
(DOCX)

S1 Text Ingenuity pathway analysis. Ingenuity systems was used to map the major pathways and processes in which miR-34c-5p is involved. The top molecular and cellular function of the miRNA predicted target genes was “cell morphology”; while the second top-associated network was “cell morphology/cellular assembly and organization/cellular development”. We used miRecords as a resource for microRNA-target interactions.
(PDF)
