# Supplementary figures and images for: Type 2 Diabetes Monocyte MicroRNA and mRNA Expression: Dyslipidemia Associates with Increased Differentiation-Related Genes but Not Inflammatory Activation
Source: PLoS One. 2015 Jun 17;10(6):e0129421. doi: 10.1371/journal.pone.0129421 (PMC4471054; doi:10.1371/journal.pone.0129421)

Suppl. Figure 2

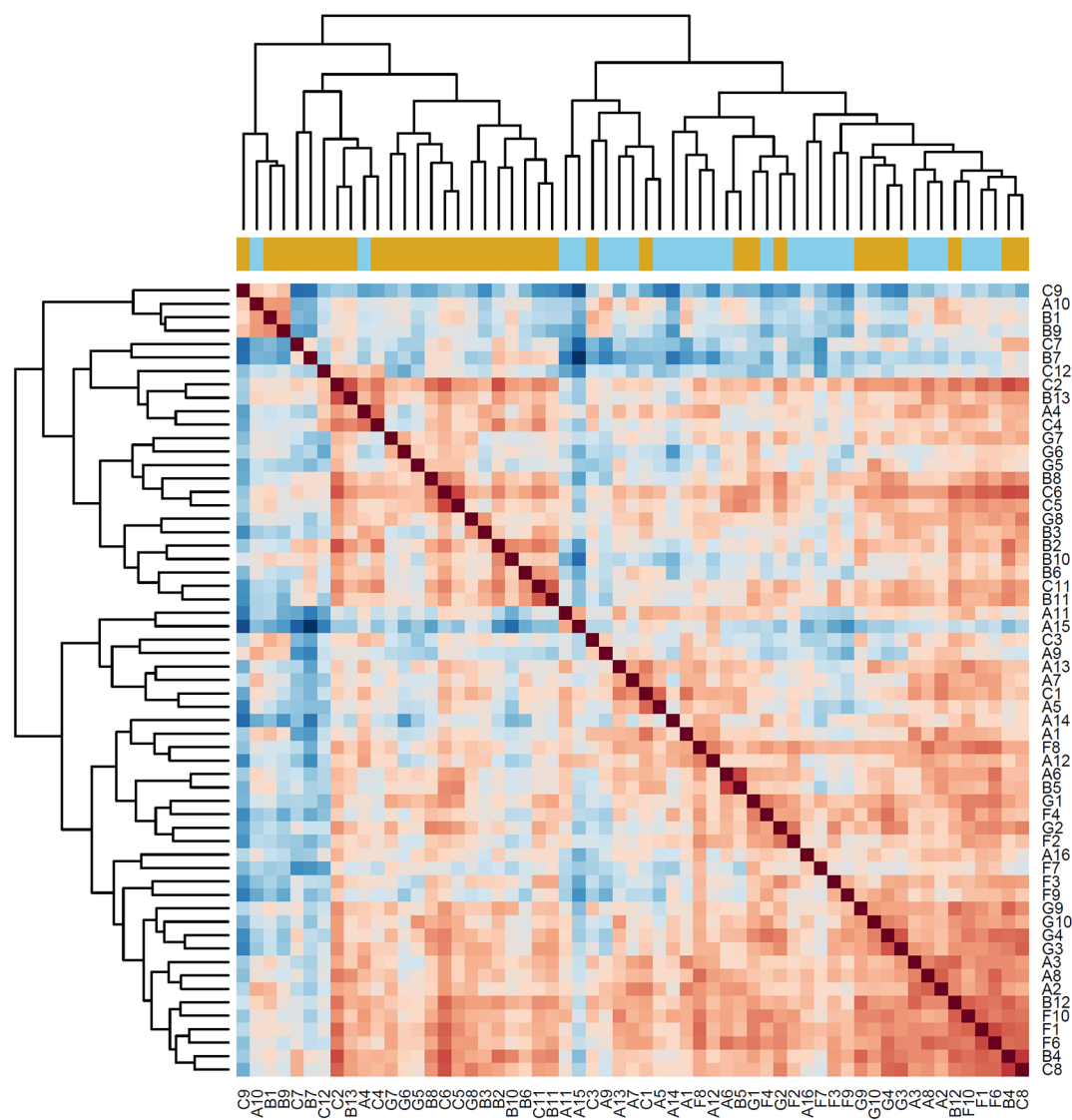

Supplement: S1 Fig — This figure shows that partial separation can be made between T2D patients and healthy controls on the basis of the 15 microRNAs identified as optimal prediction signature. Two main subject clusters were identified. The first cluster contain 24 T2D patients (yellow) and only 2 healthy controls (blue), and the second mixed cluster contains 12 patients (yellow) and 23 healthy controls (blue). (PDF) [file pone.0129421.s001.PDF]
