# Supplementary material for: Type 2 Diabetes Monocyte MicroRNA and mRNA Expression: Dyslipidemia Associates with Increased Differentiation-Related Genes but Not Inflammatory Activation
Source: PLoS One. 2015 Jun 17;10(6):e0129421. doi: 10.1371/journal.pone.0129421 (PMC4471054; doi:10.1371/journal.pone.0129421)
Supplement: S1 Table — This table shows the 142 microRNAs that were found to be differentially expressed in T2D patients compared to controls. 35% of the miRNAs were down-regulated and 65% were up-regulated. (DOCX) [file pone.0129421.s002.docx]

**S1 Table.** Differentially expressed monocyte microRNAs of T2D patients compared to non-diabetic controls of the finding cohort.

| **Up-regulated microRNAs** | | | | | | | | |
| --- | --- | --- | --- | --- | --- | --- | --- | --- |
|  | **miRNA** | **p-value** |  | **miRNA** | **p-value** |  | **miRNA** | **p-value** |
| 1 | hsa-let-7a-2* | 0.004 | 32 | hsa-miR-296-3p | 0.046 | 63 | hsa-miR-523 | 0.043 |
| 2 | hsa-let-7e | 0.016 | 33 | hsa-miR-297 | 0.012 | 64 | hsa-miR-541 | 0.002 |
| 3 | hsa-miR-10a* | 0.004 | 34 | hsa-miR-298 | 0.014 | 65 | hsa-miR-548b-5p | 0.029 |
| 4 | hsa-miR-122 | 0.010 | 35 | hsa-miR-30b* | 0.000 | 66 | hsa-miR-550 | 0.012 |
| 5 | hsa-miR-125a-3p | 0.006 | 36 | hsa-miR-30c-1* | 0.006 | 67 | hsa-miR-574-3p | 0.029 |
| 6 | hsa-miR-125a-5p | 0.033 | 37 | hsa-miR-32* | 0.006 | 68 | hsa-miR-574-5p | 0.004 |
| 7 | hsa-miR-125b-2* | 0.004 | 38 | hsa-miR-325 | 0.003 | 69 | hsa-miR-576-3p | 0.034 |
| 8 | hsa-miR-1296 | 0.000 | 39 | hsa-miR-328 | 0.041 | 70 | hsa-miR-585 | 0.002 |
| 9 | hsa-miR-130b | 0.017 | 40 | hsa-miR-329 | 0.023 | 71 | hsa-miR-595 | 0.026 |
| 10 | hsa-miR-135a* | 0.033 | 41 | hsa-miR-331-3p | 0.017 | 72 | hsa-miR-596 | 0.000 |
| 11 | hsa-miR-138 | 0.007 | 42 | hsa-miR-335 | 0.043 | 73 | hsa-miR-601 | 0.043 |
| 12 | hsa-miR-139-3p | 0.004 | 43 | hsa-miR-338-5p | 0.006 | 74 | hsa-miR-603 | 0.024 |
| 13 | hsa-miR-143* | 0.010 | 44 | hsa-miR-34c-5p | 0.002 | 75 | hsa-miR-610 | 0.021 |
| 14 | hsa-miR-184 | 0.015 | 45 | hsa-miR-370 | 0.033 | 76 | hsa-miR-617 | 0.022 |
| 15 | hsa-miR-185 | 0.007 | 46 | hsa-miR-371-3p | 0.000 | 77 | hsa-miR-625 | 0.005 |
| 16 | hsa-miR-185* | 0.002 | 47 | hsa-miR-376a* | 0.021 | 78 | hsa-miR-629 | 0.009 |
| 17 | hsa-miR-187* | 0.004 | 48 | hsa-miR-423-5p | 0.024 | 79 | hsa-miR-638 | 0.021 |
| 18 | hsa-miR-1908 | 0.035 | 49 | hsa-miR-432* | 0.000 | 80 | hsa-miR-642 | 0.012 |
| 19 | hsa-miR-193a-5p | 0.021 | 50 | hsa-miR-433 | 0.036 | 81 | hsa-miR-647 | 0.030 |
| 20 | hsa-miR-193b* | 0.004 | 51 | hsa-miR-449a | 0.002 | 82 | hsa-miR-658 | 0.000 |
| 21 | hsa-miR-194* | 0.002 | 52 | hsa-miR-483-5p | 0.000 | 83 | hsa-miR-659 | 0.003 |
| 22 | hsa-miR-195 | 0.025 | 53 | hsa-miR-488 | 0.021 | 84 | hsa-miR-675 | 0.029 |
| 23 | hsa-miR-198 | 0.002 | 54 | hsa-miR-492 | 0.021 | 85 | hsa-miR-760 | 0.035 |
| 24 | hsa-miR-200b* | 0.004 | 55 | hsa-miR-497 | 0.008 | 86 | hsa-miR-765 | 0.002 |
| 25 | hsa-miR-202 | 0.022 | 56 | hsa-miR-498 | 0.002 | 87 | hsa-miR-877 | 0.002 |
| 26 | hsa-miR-210 | 0.003 | 57 | hsa-miR-501-3p | 0.017 | 88 | hsa-miR-885-3p | 0.027 |
| 27 | hsa-miR-215 | 0.017 | 58 | hsa-miR-505* | 0.012 | 89 | hsa-miR-888* | 0.032 |
| 28 | hsa-miR-219-2-3p | 0.044 | 59 | hsa-miR-515-3p | 0.012 | 90 | hsa-miR-92b* | 0.036 |
| 29 | hsa-miR-22* | 0.047 | 60 | hsa-miR-516b | 0.018 | 91 | hsa-miR-936 | 0.000 |
| 30 | hsa-miR-24-1* | 0.046 | 61 | hsa-miR-518c* | 0.000 | 92 | hsa-miR-96* | 0.033 |
| 31 | hsa-miR-25* | 0.012 | 62 | hsa-miR-519d | 0.002 | 93 | hsa-miR-99b* | 0.001 |

| **Down-regulated microRNAs** | | | | | | | | |
| --- | --- | --- | --- | --- | --- | --- | --- | --- |
|  | **miRNA** | **p-value** |  | **miRNA** | **p-value** |  | **miRNA** | **p-value** |
| 1 | hsa-let-7a | 0.002 | 18 | hsa-miR-302b* | 0.018 | 34 | hsa-miR-548c-3p | 0.010 |
| 2 | hsa-let-7c* | 0.018 | 19 | hsa-miR-302c* | 0.043 | 35 | hsa-miR-556-3p | 0.019 |
| 3 | hsa-let-7g | 0.017 | 20 | hsa-miR-30a | 0.047 | 36 | hsa-miR-586 | 0.002 |
| 4 | hsa-miR-144* | 0.032 | 21 | hsa-miR-31* | 0.013 | 37 | hsa-miR-607 | 0.032 |
| 5 | hsa-miR-148b | 0.019 | 22 | hsa-miR-326 | 0.012 | 38 | hsa-miR-616* | 0.049 |
| 6 | hsa-miR-150 | 0.008 | 23 | hsa-miR-374a | 0.021 | 39 | hsa-miR-625* | 0.004 |
| 7 | hsa-miR-154 | 0.029 | 24 | hsa-miR-410 | 0.042 | 40 | hsa-miR-628-3p | 0.012 |
| 8 | hsa-miR-191 | 0.006 | 25 | hsa-miR-424 | 0.042 | 41 | hsa-miR-649 | 0.040 |
| 9 | hsa-miR-19a | 0.035 | 26 | hsa-miR-450b-5p | 0.019 | 42 | hsa-miR-651 | 0.029 |
| 10 | hsa-miR-19a* | 0.009 | 27 | hsa-miR-486-5p | 0.018 | 43 | hsa-miR-652 | 0.000 |
| 11 | hsa-miR-20b | 0.008 | 28 | hsa-miR-491-3p | 0.009 | 44 | hsa-miR-656 | 0.003 |
| 12 | hsa-miR-216b | 0.007 | 29 | hsa-miR-494 | 0.010 | 45 | hsa-miR-662 | 0.045 |
| 13 | hsa-miR-223 | 0.000 | 30 | hsa-miR-509-3-5p | 0.001 | 46 | hsa-miR-720 | 0.002 |
| 14 | hsa-miR-223* | 0.033 | 31 | hsa-miR-520f | 0.035 | 47 | hsa-miR-9* | 0.018 |
| 15 | hsa-miR-26a | 0.007 | 32 | hsa-miR-532-5p | 0.049 | 48 | hsa-miR-921 | 0.023 |
| 16 | hsa-miR-26b | 0.046 | 33 | hsa-miR-548a-3p | 0.009 | 49 | hsa-miR-943 | 0.002 |
| 17 | hsa-miR-301b | 0.023 |  |  |  |  |  |  |
