# Supplementary material for: Type 2 Diabetes Monocyte MicroRNA and mRNA Expression: Dyslipidemia Associates with Increased Differentiation-Related Genes but Not Inflammatory Activation
Source: PLoS One. 2015 Jun 17;10(6):e0129421. doi: 10.1371/journal.pone.0129421 (PMC4471054; doi:10.1371/journal.pone.0129421)
Supplement: S2 Table — Values represent the means and standard deviations of normalized Ct values (Ct gene/Ct reference gene ABL) by the ΔΔCt. Genes are given in the order of the cluster diagram given in this paper. The table shows significantly reduced expression levels of many of the classical inflammatory cluster A and B genes in the Ecuadorian non-diabetic controls compared to the Dutch controls. Cluster C genes were largely unaltered in the monocytes of the Ecuadorian group. Of note, these analyses were performed in the same time period to exclude technical variability. (DOCX) [file pone.0129421.s003.docx]

**S2 Table .** Monocyte gene expression of the Ecuadorian non-diabetic general population controls vs the Dutch general population controls.

|  | **Gene** | **Ecuadorian** | | **Dutch** | | **p-Value** |
| --- | --- | --- | --- | --- | --- | --- |
|  |  | **NDC** | | **NDC** | |  |
|  |  | Mean | SD | Mean | SD |  |
| **CLUSTER A** | **IL6** | ***0.01*** | 0.6 | 0.30 | 0.7 | ******** |
|  | **TNF** | ***2.14*** | 2.5 | 2.65 | 2.6 | ns |
|  | **IL1B** | ***9.25*** | 85.8 | 70.95 | 107.1 | ********* |
|  | **CCL2** | 0.40 | 0.8 | 0.71 | 1.1 | ******* |
|  | **CCL20** | ***0.05*** | 1 | 0.49 | 1.1 | ******** |
|  | **TNFAIP3** | ***1.91*** | 5.3 | 5.78 | 6.3 | ********* |
|  | **PDE4B** | ***3.13*** | 6.4 | 7.81 | 8 | ********* |
|  | **DUSP2** | 1.49 | 2.1 | 2.50 | 2.2 | ******** |
|  | **PTGS2** | 3.22 | 3 | 2.57 | 2.2 | ns |
|  | **ATF3** | ***1.62*** | 1.6 | 2.66 | 1.6 | ******** |
| **CLUSTER B** | **CDC42** | 1.12 | 0.7 | 1.13 | 1.4 | ns |
|  | **PTX3** | ***0.52*** | 1.8 | 1.50 | 2 | ******** |
|  | **CXCL2** | ***0.72*** | 7.6 | 5.55 | 8.5 | ********* |
|  | **STX1A** | ***0.01*** | 0.1 | 0.04 | 0.1 | ******** |
|  | **NAB2** | 0.29 | 0.5 | 0.41 | 0.5 | ns |
|  | **EMP1** | ***0.49*** | 1.1 | 1.04 | 1.3 | ******** |
|  | **BCL2A1** | 18.85 | 21 | 25.68 | 67.2 | ns |
|  | **PTPN7** | 0.18 | 0.1 | 0.17 | 0.1 | ns |
| **CLUSTER C** | **FABP5** | 16.41 | 37.5 | 19.51 | 117.2 | ns |
|  | **MAPK6** | 5.69 | 10.4 | 7.80 | 37.1 | ns |
|  | **HSPA1A;HSPA1B** | 19.65 | 22.5 | 16.45 | 52.3 | ns |
|  | **DHRS3** | 0.06 | 0 | 0.07 | 0 | ns |
|  | **CD9** | 2.78 | 2.9 | 2.10 | 2.7 | ns |

***Note.*** *Statistical significance: *p< .05;**p< .01; ***p< .001*

**S2 Table.** *Monocyte gene expression of the Ecuadorian non-diabetic controls (n=39) vs. the Dutch non-diabetic controls (n=74) of the same ages (age range 30-70) and gender distribution (over-representation of females).*

Values represent the means and standard deviations of normalized Ct values (Ct gene/Ct reference gene ABL) by the ΔΔCt method. Genes are given in the order of the cluster diagram (see Fig. 1).
