# Supplementary material for: Type 2 Diabetes Monocyte MicroRNA and mRNA Expression: Dyslipidemia Associates with Increased Differentiation-Related Genes but Not Inflammatory Activation
Source: PLoS One. 2015 Jun 17;10(6):e0129421. doi: 10.1371/journal.pone.0129421 (PMC4471054; doi:10.1371/journal.pone.0129421)
Supplement: S1 Text — Ingenuity systems was used to map the major pathways and processes in which miR-34c-5p is involved. The top molecular and cellular function of the miRNA predicted target genes was “cell morphology”; while the second top-associated network was “cell morphology/cellular assembly and organization/cellular development”. We used miRecords as a resource for microRNA-target interactions. (PDF) [file pone.0129421.s004.PDF]

Analysis Name: Target prediction miR-34c-5p - 2014-04-15 05:38 PM
Analysis Creation Date: 2014-04-15
Build version: 302937
Content version: 18488943 (Release Date: 2014-03-23)

Analysis settings

View

Reference set: Ingenuity Knowledge Base (Genes Only)

Relationship to include: Direct and Indirect

Includes Endogenous Chemicals

Optional Analyses: My Pathways My List

Filter Summary:

Consider only relationships where

confidence = Experimentally Observed

Cutoff:

(c) 2000-2014 Ingenuity Systems, Inc. All rights reserved.

Top Canonical Pathways

Name	p-value	Ratio
Glioblastoma Multiforme Signaling	1,56E-08	43/168 (0,256)
Dopamine-DARPP32 Feedback in cAMP Signaling	1,43E-07	44/187 (0,235)
STAT3 Pathway	1,51E-07	26/80 (0,325)
Axonal Guidance Signaling	2,21E-07	89/487 (0,183)
Molecular Mechanisms of Cancer	2,91E-07	74/388 (0,191)

Top Upstream Regulators

Upstream Regulator	p-value of overlap	Predicted Activation State
mir-34	1,88E-23	
beta-estradiol	4,09E-12	
miR-34a-5p (and other miRNAs w/seed GGCAGUG)	2,25E-11	
TGFB1	1,15E-10	
AGT	1,82E-10	

(c) 2000-2014 Ingenuity Systems, Inc. All rights reserved.

INGENUITY® SYSTEMS

Top Diseases and Bio Functions

Diseases and Disorders

Name	p-value	# Molecules
Cancer	4,04E-35 - 9,14E-06	1898
Developmental Disorder	4,32E-21 - 3,20E-06	414
Cardiovascular Disease	4,32E-17 - 5,45E-06	415
Neurological Disease	7,69E-12 - 4,12E-06	570
Gastrointestinal Disease	1,36E-11 - 5,38E-06	800

Molecular and Cellular Functions

Name	p-value	# Molecules
Cell Morphology	2,44E-19 - 9,35E-06	586
Cell Death and Survival	2,62E-19 - 9,44E-06	756
Molecular Transport	2,58E-18 - 3,81E-06	533
Cellular Movement	1,24E-17 - 5,92E-06	489
Cellular Development	2,24E-16 - 9,48E-06	717

(c) 2000-2014 Ingenuity Systems, Inc. All rights reserved.

Physiological System Development and Function

Name	p-value	# Molecules
Organismal Survival	2,32E-27 - 4,14E-06	588
Behavior	2,21E-23 - 9,31E-06	294
Nervous System Development and Function	2,80E-19 - 9,35E-06	541
Organismal Development	4,74E-18 - 7,90E-06	724
Tissue Morphology	7,25E-18 - 9,35E-06	586

(c) 2000-2014 Ingenuity Systems, Inc. All rights reserved.

Top Tox Functions

Assays: Clinical Chemistry and Hematology

Name	p-value	# Molecules
Increased Levels of Hematocrit	2,27E-03 - 2,27E-03	22
Increased Levels of Red Blood Cells	2,27E-03 - 7,34E-02	22
Increased Levels of Alkaline Phosphatase	6,81E-03 - 1,20E-01	18
Increased Levels of Albumin	6,10E-02 - 5,36E-01	6
Increased Levels of Blood Urea Nitrogen	7,77E-02 - 7,77E-02	4

Cardiotoxicity

Name	p-value	# Molecules
Heart Failure	4,01E-07 - 6,41E-01	61
Cardiac Arrythmia	1,17E-06 - 6,41E-01	50
Tachycardia	1,52E-06 - 1,20E-01	26
Cardiac Arteriopathy	5,44E-06 - 6,41E-01	63
Congenital Heart Anomaly	8,43E-05 - 1,00E00	38

(c) 2000-2014 Ingenuity Systems, Inc. All rights reserved.

Hepatotoxicity

Name	p-value	# Molecules
Liver Proliferation	9,72E-07 - 2,32E-01	49
Liver Hepatomegaly	2,48E-06 - 2,48E-06	25
Liver Inflammation/Hepatitis	9,62E-06 - 4,01E-01	60
Liver Steatosis	9,62E-06 - 4,01E-01	53
Liver Hypoplasia	3,31E-04 - 3,31E-04	14

Nephrotoxicity

Name	p-value	# Molecules
Renal Necrosis/Cell Death	3,37E-09 - 4,63E-01	96
Kidney Failure	2,40E-05 - 1,00E00	45
Renal Inflammation	2,50E-05 - 1,00E00	39
Renal Nephritis	2,50E-05 - 1,00E00	39
Glomerular Injury	6,18E-05 - 5,03E-01	33

Top Regulator Effect Networks

(c) 2000-2014 Ingenuity Systems, Inc. All rights reserved.

Top Networks

ID	Associated Network Functions	Score
1	Digestive System Development and Function, Infectious Disease, Organ Morphology	35
2	Cell Morphology, Cellular Assembly and Organization, Cellular Development	34
3	Cellular Development, Embryonic Development, Organ Development	34
4	Behavior, Molecular Transport, Cell Morphology	32
5	Connective Tissue Disorders, Dental Disease, Developmental Disorder	32

Top Tox Lists

Name	p-value	Ratio
Renal Necrosis/Cell Death	1,25E-07	96/472 (0,203)
Liver Proliferation	7,34E-06	49/216 (0,227)
Cardiac Fibrosis	1,8E-05	42/181 (0,232)
Cardiac Hypertrophy	2,94E-05	72/373 (0,193)
VDR/RXR Activation	2,59E-04	21/78 (0,269)

(c) 2000-2014 Ingenuity Systems, Inc. All rights reserved.

Top My Lists

Name	p-value	Ratio

Top My Pathways

Name	p-value	Ratio

Top Molecules

This analysis has no expression values.

(c) 2000-2014 Ingenuity Systems, Inc. All rights reserved.
